# The effects of the COVID-19 pandemic on patients with lysosomal storage disorders in Israel

**DOI:** 10.1186/s13023-021-02007-9

**Published:** 2021-09-08

**Authors:** Eyal Kristal, Ben Pode-Shakked, Guy Hazan, Ehud Banne, Galina Ling, Odeya David, Eilon Shany, Annick Raas-Rothschild, Yair Anikster, Katya Kneller, Eli Hershkovitz, Yuval E. Landau, Ronen Spiegel, Yoav Zehavi, Orna Staretz-Chacham

**Affiliations:** 1 Pediatric Ambulatory Day Unit, Soroka Medical Center, Beer Sheva, Israel; 2 Metabolic Clinic, Soroka University Medical Center, Faculty of Health Sciences, Ben-Gurion University, Beer Sheva, Israel; 3 Sackler Faculty of Medicine, Tel Aviv University, Tel-Aviv, Israel; 4 Edmond and Lily Safra Children’s Hospital, Sheba Medical Center, Tel-Hashomer, Israel; 5 Pediatric D Department, Soroka Medical Center, Beer Sheva, Israel; 6 The Rina Mor Institute of Medical Genetics, Wolfson Medical Center, Holon, Israel; 7 Neonatology Unit, Soroka Medical Center, Beer Sheva, Israel; 8 Metabolic Disease Unit, Schneider Children’s Medical Center of Israel, Tel Aviv University, Beer Sheva, Israel; 9 Department of Pediatrics B, Metabolic Service, Emek Medical Center, Afula, Israel; 10 Ruth and Bruce Rappaport Faculty of Medicine, Technion-Israel Institute of Technology, Haifa, Israel; 11 Metabolic Clinic, Soroka Medical Center, Beer Sheva, Israel

**Keywords:** Lysosomal storage disorder, COVID-19, Enzyme replacement therapy

## Abstract

**Background:**

Severe acute respiratory syndrome coronavirus 2 (SARS CoV-2) is the causative agent of the current COVID-19 pandemic. Lysosomal storage disorders (LSD) comprise of 70 inherited inborn errors of metabolism. Affected individuals suffer from multi-systemic involvement with variable severity and rate of disease progression between different diseases. Some of the LSDs have established treatments, whether parenteral or oral therapies. The full impact of the COVID-19 pandemic together with the lockdown on the wellbeing and medical management of patients with rare diseases, such as LSDs, is widely unknown. Herein, we describe the effects of the COVID-19 pandemic and its associated mandatory home lockdown on patients with LSDs in Israel.

**Results:**

We present a prospective multi-center questionnaire study including 48 LSD patients from four medical centers in Israel. The study objective was to assess the impact of the COVID-19 pandemic restrictions on individuals with LSDs in Israel, as reported by their caregivers. Secondary objectives were to assess the morbidity from SARS CoV-2 in LSD patients and the impact of changes in mood and behavior on compliance to treatment and to assess the relationship between changes in mood to changes in cognition and behavior. Thirty one of 38 patients (82%) who received any kind of regular treatment did not miss treatments. Among patients receiving enzyme replacement therapy (ERT) in the in-hospital setting, 5 patients (20%) experienced treatment disruptions. Four patients had tested positive for SARS-Cov-2 virus infection by PCR. Seven out of the 48 patients (14%) described mood changes with cognitive and motor deterioration during the home quarantine.

**Conclusions:**

We observed high rates of treatment adherence and low morbidity through the COVID-19 pandemic in patients with LSDs in Israel. LSDs patients can be a model for patients with complex chronic diseases requiring routine treatments and surveillance during a pandemic or other disruption of daily routine.

## Background

Lysosomal storage disorders (LSD) comprise of 70 inherited inborn errors of metabolism. While individually rare, as a group their incidence is 1:5000 live births [1]. The lysosome is responsible in the cells for macromolecule catabolism, recycling and signaling. Mutations in lysosomal genes lead to impaired encoded protein and result in lysosomal malfunction and the gradual accumulation of substrates inside the lysosome [1]. Affected individuals suffer from multi-systemic involvement with variability in severity and rate of disease progression among the different diseases. Some of the LSDs have established treatments, whether stem cell transplantation, parenteral, such as enzyme replacement therapy (ERT) or oral treatments, such as substrate reduction therapy or chaperone therapy. Additionally, all affected patients need supportive therapy such as physical therapy, etc. Both ERT and supportive therapy are ambulatory treatments provided either in-hospital, out-patient clinic or at home setting.

In mid-December 2019, a new strain of betacoronavirus emerged as the causative pathogen of pneumonia in the city of Wuhan, Hubei Province, China. The new strain rapidly spread resulting in a worldwide pandemic [2–4]. In February 2020, the World Health Organization (WHO) designated the new virus as severe acute respiratory syndrome coronavirus 2 (SARS-CoV-2) and the ensuing disease as coronavirus disease 2019 (COVID-19) [5]. In March 2020, the WHO labeled it a global pandemic. COVID-19 soon spread across the globe, with more than 100,000,000 patients diagnosed by January 2021. In February 2020, Israel diagnosed its first patient with COVID-19. In March–May 2020, Israel implemented stringent social distancing and quarantine (lockdown) measures, resulting in low mortality and morbidity rates. On March 12th, schools and universities were closed, followed by a nationwide lockdown with a strict social distancing policy starting on March 19th [6, 7]. Nearly all flights to Israel were closed by early March. Part of these measures were discontinued in early May as a result of a decline in SARS-CoV-2 morbidity. As in the first lockdown, a second lockdown was declared on September 18th and relieved a month later, on October 18th. The third lockdown took effect in late December 2020 and lasted until early February 2021. If compared to other places around the globe- in Western countries, whether U.S.A or Europe those strict decisions were delayed, leading to higher mortality and morbidity [7]. On December 19th, 2020, Israel launched its adult coronavirus vaccination, achieving a 60% vaccination rate (after two doses) in adults by late March 2021 [8]. The mortality from COVID-19 in Israel so far was 71:100,000 cases (0.8% of affected cases).

In Israel, the Ministry of Health bears national responsibility for ensuring the health of the population. All citizens are covered by public Health Maintenance Organization (HMO) plans that enables them for highly extended treatment as needed not-related to their socioeconomic situation. These include availability to non-specific therapies such as rehabilitation, consultations with specialists and also access to high-cost drugs for everyone, if included in the Israeli health basket, or according to different HMO plans when these drugs are not included. Individuals with certain chronic disorders are especially vulnerable to severe effects of SARS-CoV-2 infection with significant morbidity and mortality [9, 10]. We hypothesized that due to the special challenges of individuals living with chronic diseases, the COVID-19 pandemic related restrictions impacted their wellbeing as well as their medical conditions.

The objective of this study was to assess the impact of the COVID-19 pandemic restrictions on individuals with LSDs in Israel, as reported by their caregivers. Secondary objectives were to assess the impact of changes in mood and behavior on compliance to treatment and to assess the relationship between changes in mood to changes in cognition and behavior.

## Results

Eight out of 15 physicians responded with information on a total of 48 patients treated in four different medical centers in Israel. The patient cohort comprised of 29 males (60%) and 19 females (40%), ages between 1 to 42 years, with 46 of the patients (95%) under 18 years of age (Table 1) and a median age of 6.5 years (range 41). Patients included in the study were affected by the following diseases: 27 patients with mucopolysaccharidosis (MPS) (56%): 1 MPS type I (Hurler syndrome), 2 MPS type II (Hunter syndrome), 8 MPS type IIIA (Sanfilippo A syndrome), 14 MPS type IVA (Morquio syndrome), 2 MPS type VI (Maroteaux-Lamy syndrome); 7 Pompe disease (15%), 6 Niemann Pick type C disease (12%); 2 Gaucher type 1 disease (4%); 2 neuronal ceroid lipofuscinosis (NCL) (4%; of these one with NCL5 and another with NCL6); 1 Niemann Pick type A disease (2%); 1 cystinosis (2%); 1 I cell disease (2%); and 1 Lysosomal acid lipase deficiency (2%).

**Table 1:** Descriptive analysis for questionnaire data

Questionnaire data	No. ( %)
Total	48 (100)
Males (%)	29 (60)
No. (%) of patients treated with ERT* *or other parenteral*	*28 (58.3)*
No.(%) of patients that missed ERT or parenteral treatments during COVID-19 pandemic	7 (25)
No. (%) of patients that were under the age 18 years during COVId-19 pandemic	46 (95)
No. (%) of patients reporting mood deterioration during COVID-19 pandemic	7 (14.6)
No. (%) of patients reporting increased aggressiveness during COVID-19 pandemic	7 (14.6)
No. (%) of patients reporting cognitive function decline during COVID-19 pandemic	8 (16.7)
No. (%) of patients reporting motor function decline during COVID-19 pandemic	11 (23.9)

*ERT enzyme replacement therapy

Four patients had tested positive for SARS-Cov-2 virus infection by PCR, all four were patients diagnosed with Sanfilippo A and two of them were siblings.

Regarding treatment modalities of underlying LSD, 26 patients (55%) are treated with intravenous ERT, and 15 patients (31%) are on different oral therapies, such as chaperone therapy or supportive, while 7 patients (14%) have no regular therapies.

Regarding ERT treated patients, before the COVID-19 outbreak, 24 patients (92%) were regularly receiving intravenous infusions in the hospital and two patients (8%) were on home-therapy. Due to the pandemic, two additional patients were transferred to home therapy.

Thirty one of 38 patients (82%) who received any kind of regular treatment did not miss treatments. Among patients receiving ERT in the in-hospital setting, 5 patients (20%) experienced treatment disruptions: three missed an intravenous infusion due to their parents' fear of arrival to the hospital, one patient missed an intravenous infusion due to logistic issues in his medical center and one patient missed an intravenous infusion due to low adherence, regardless of the COVID-19 pandemic.

Seven out of the 48 patients (14.5%) described mood changes with cognitive and motor deterioration during the home quarantine (Table 1).

Fourteen patients of our cohort were Morquio patients (29%) of whom 2 patients missed one ERT (14%) infusion and one patient was not on ERT not related to COVID-19. Nine Morquio patients (64%) reported missing physiotherapy treatments during the quarantine times, due to difficulties in approaching these services. However, since these patients' compliance with the additional treatments was low even before the COVID-19 era, this did not have major effects on the patients.

The subgroup of patients who missed ERT is small (n = 7), so these findings should be considered accordingly and require confirmation in larger cohorts. Patients with mood deterioration had 76 times more aggressiveness, 37 times more cognitive function decline and 12.8 times more motor function deterioration (all with Pv < 0.001) (Table 2).

**Table 2:** Univariate analysis (by chi square) for symptoms associated with mood deterioration, respectively, during pandemic

Symptom onset	Association with mood deterioration (N = 48)	
	Pv	OR (CI)
Aggressiveness (N = 48)	< 0.001	76 (6.7–855.9)
Cognitive function decline (N = 48)	< 0.001	37 (5.1–269.7)
Motoric function deterioration (N = 46)	0.001	12.8 (2.4–68.4)

## Discussion

This study on LSD patients showed relatively undisrupted care during the COVID-19 pandemic in Israel, despite significant psychological stress of these patients as expressed by aggressiveness, cognitive function decline and motoric function deterioration that were related to general mood deterioration. Although, when compared to previous studies on LSD patients such as Sanfilippo patients [11, 12], it is difficult to assess whether aggressiveness or cognitive deterioration were indeed due to the stress accompanying the lockdown or actually part of the natural history of the disease, the caregivers descriptions and later on their reports on the improvement with lockdown relieve suggest that part of the above deterioration was indeed expedited due to the psychological stress.

The routine management of patients affected with rare inborn errors of metabolism with multisystemic involvement is complex and carries a significant burden on the patients and their caregivers, all the more so during a global pandemic.

Regular drug supply and deliveries were ensured all over Israel. No interruption or modification occurred for patients receiving oral therapy, except for Amborxol treatment for Gaucher patients due to supply problems.

In our cohort we observed overall high adherence to ERT, even though most of the patients receive treatment in the in-hospital setting and not at home. All patients received therapy in hospitals in which COVID-19 patients were also admitted. In all cases the treatment was in areas well-separated between COVID-19 and non-COVID-19 patients. Sechi et al. described the impact of the COVID-19 related healthcare crisis on treatments for patients with LSDs in Italy [13]. No interruption or modification occurred for patients receiving only oral therapy. Regarding patients on ERT, all patients who were already on home-therapy continued their infusions regularly, while among patients receiving ERT in the hospital nearly half (49%) experienced treatment disruptions, mainly because of parental fear of infection and due to reorganization of the infusion center. The higher rate of ERT adherence in our cohort can be explained by lower mortality rate from COVID-19 in Israel compared to Italy (Johns Hopkins Coronavirus Resource Center) combined with the Israeli healthcare system response to the pandemic which excluded disruptions to chronic treatment including ERT for LSDs patients. An important issue contributing to this result is the secluded areas these ambulatory patients are treated in, which reduced families’ fear of getting to the hospital during the pandemic. Of note, our results were comparable with those recently reported in a cohort of Gaucher Disease patients in Spain, in whom dose interruptions in patients treated in in-patient settings were reported in 25% of patients [14].

As could be expected most of the patients reported with mood changes and cognitive decline suffer from neurodegenerative diseases, five are affected by Sanfilippo A and one is affected by NCL-6, that have more profound baseline neurological symptoms. The questionnaires in those patients were filled by their treating physicians with the help of their caregivers, who all noted that the prolonged home quarantine may be the reason for the accelerated deterioration of the clinical and mental status of our patients. Although not all patients with LSDs receive regular paramedical treatments, the reasons for missed treatments can be decreased availability of paramedical treatment during the COVID-19 pandemic from medical side, and the parents’ perception of those treatments as less significant during the COVID-19 pandemic, from the patients'/families' side. Patients with LSDs may be considered at high risk of developing severe complications in case of SARS-CoV-2 infection, especially, since they tend to have a multisystem involvement. From our survey data, among 48 reported patients, only 4 patients (6.2%) had tested positive for SARS-CoV-2, and all of them had mild disease with no requirement for hospital admission. Compared to the general Israeli population, the infection rate among LSDs patients is higher (4.4% based on the Israeli Ministry of Health data) but the mortality and morbidity rate is significantly lower. While this might be the result of the relatively small patient cohort, this finding may actually be supported by a study of transcriptomic analyses performed on MPS patients' cell lines by Pierzynowska et al. that showed that MPS patients' cells may have lower susceptibility to infection by SARS-CoV-2 due to specific changes in expression of genes coding for proteins involved in interactions with viral proteins [15]. Another important point is the vast majority of the described Israeli cohort includes younger patients which could partially explain the mild disease, as occurs also in the general pediatric population in Israel. Fierro et al. described a cohort of 181 patients with Gaucher disease (GD) and SARS-CoV-2 infection In New York [16] and they concluded that GD patients do not appear to have an increased risk for severe SARS-CoV-2 infection. The authors also raise the possibility that lysosomal dysfunction underlying GD pathophysiology, may somehow protect or otherwise modify responses to SARS-CoV-2 infection, and this could also be the reason in other LSD patients, as described here.

Finally, an important and often overlooked aspect of the holistic care of children and adults affected with inborn errors of metabolism, and specifically LSDs, is the psychological state and wellbeing. Recently, Fiumara et al. [17] evaluated the psychological impact of the COVID-19 pandemic on a cohort of 15 LSD patients in Italy. While their results did not reach statistical significance when compared to age-matched controls, an important finding universally shared by all participants was the reportedly increased feelings of anxiety, worry and uncertainty. As the investigators noted, home reclusion further contributed to feelings of being excluded, known all too well to individuals affected by rare chronic disease. While our study was not designed to evaluate specific patient perceptions and feelings, the overall added psychological burden associated with the global pandemic was reflected in the reported mood changes associated with motor and cognitive deterioration during the prolonged home lockdown. These can be compared to the general population as reported by Terry et al. [18] that in a cohort of 1062 participants showed results that confirmed significant mood disturbance during the period of COVID-19 restrictions, representing increased risk of psychopathology, more significant in the younger population under 25 years. Terry et al. has also demonstrated that those with higher education scored lower for depression and fatigue and higher for vigour compared to those with lower education. This could partially relate to the reports in our study of most severe psychological impact on the neurodegenerative affected patients.

Our study has several limitations. First, the size of the cohort is limited, although a cohort of 50 participants in rare diseases is regarded differently than in other areas. Further, our results reflect the Israeli organization of the Healthcare System that at the time of the study was not yet faced with massive loads of patients. Second, the study did not include a control cohort, such as healthy individuals or patients harboring other chronic conditions necessitating both routine in-patient and out-patient care. Subsequently, we compared our results with published papers of healthy population around the globe, and discussed the results in that context. Further studies, evaluating larger cohorts of patients and comparing them to relevant control cohorts could serve to reaffirm our findings and shed new light on the medical and psychological effects of pandemic-associated quarantine in patients with rare inborn errors of metabolism and other rare inherited diseases.

## Conclusion

We observed high rates of treatment adherence and low morbidity through the COVID-19 pandemic in a cohort of 48 patients with LSDs in Israel. As this pandemic created a new daily routine in all aspects of life, it is still unclear what will be the cumulative outcomes in COVID-19 infected patients and in uninfected patients with chronic disease for whom treatment was interrupted. LSDs patients can be a model for patients with complex chronic diseases requiring routine treatments and surveillance during a pandemic or other disruption of daily routine.

## Materials and methods

### Study design

This was a prospective multi-center questionnaire study conducted between the two lockdowns that started at March-October 2020. The study was approved by the local ethics committee of the correspondent author. Physicians caring for individuals living with LSDs in Israel were reached via e-mail and phone calls and asked to fill a questionnaire. The physicians were approached from a list of known Israeli metabolic physicians. The questionnaires included data regarding the following variables: demographic data, including patient age and gender; general clinical characteristics, including the specific IEM diagnosis, and regular therapeutic interventions (whether oral medication, ERT or research-based compounds); as well as questions focused on patients’ adherence to the different treatment modalities, whether or not the patient had missed treatment appointments, the number of missed treatment appointments and the reasons behind them, as well as the effects of the prolonged lockdown on their motor function (e.g. difficulty walking), cognitive abilities (e.g. changes in vocabulary, comprehension, etc.), mental wellbeing (e.g. mood changes, feelings of depression, unwillingness to do things or get out of bed) and/or behavior (e.g. increased aggressiveness). The physicians filled the questionnaire based on their knowledge of the patients and with the help of their caregivers.

### Statistical analysis

All statistical analyses were conducted using SPSS Statistics for Windows, version 25.0 (SPSS Inc., Chicago, USA). For descriptive analysis, central tendency and dispersion values were calculated. For univariable analysis chi square test was used.

## Data Availability

The datasets used and/or analyzed during the current study are available from the corresponding author for reasonable request.

## Abbreviations

COVID-19: Coronavirus disease 2019
ERT: Enzyme replacement therapy
GD: Gaucher disease
HMO: Health maintenance organization
IEM: Inborn error of metabolism
LSD: Lysosomal storage disorders
MPS: Mucopolysaccharidosis
NCL: Neuronal ceroid lipofuscinosis
PCR: Polymerase chain reaction
SARS-CoV-2: Severe acute respiratory syndrome coronavirus 2
WHO: World Health Organization
